# Organizational attributes of interprofessional primary care for adults with intellectual and developmental disabilities in ontario, Canada: a multiple case study

**DOI:** 10.1186/s12875-021-01502-z

**Published:** 2021-07-22

**Authors:** Nicole Bobbette, Rosemary Lysaght, Hélène Ouellette-Kuntz, Joan Tranmer, Catherine Donnelly

**Affiliations:** 1 School of Rehabilitation Therapy, Faculty of Health Sciences, Queen’s University, Kingston, Canada; 2 Department of Public Health Sciences & Psychiatry (Division of Developmental Disabilities), Faculty of Health Sciences, Queen’s University, Kingston, Canada; 3 School of Nursing, Faculty of Health Sciences & Institute for Clinical Evaluative Sciences, Queen’s University, Kingston, Canada

**Keywords:** Primary care, Interprofessional care, Health care teams, Intellectual and developmental disabilities

## Abstract

**Background:**

Access to high-quality primary care has been identified as a pressing need for adults with intellectual and developmental disabilities (IDD). Adults with IDD live with complex physical and mental health conditions, use health services differently than the general population and continue to face challenges when accessing health services. Interprofessional primary care teams offer comprehensive and coordinated approaches to primary care delivery and are well-positioned to address the needs of adults with IDD and other vulnerable populations. Although interprofessional primary care teams are recommended, there is currently limited understanding of how interprofessional care is delivered and how access to a team of providers improves the health of this population. The aim of this paper is to describe the organizational attributes of interprofessional primary care for adults with IDD within and across models of team-based care in one local health service context.

**Methods:**

A multiple case study was conducted with five interprofessional primary care teams in Ontario, Canada. Multiple methods were used to generate data including: a survey, document review, electronic medical record report and qualitative interviews. Pattern matching was the primary analytic approach for the within and across case analysis.

**Results:**

Adults with IDD were found to be a small part of the patient population served and this group was poorly identified in three of five teams. Key organizational attributes that support the delivery of interprofessional primary care for adults with IDD were identified. Two examples of targeted programs of care for this group were also found. Despite the presence of interprofessional health providers in all teams, there were limited organizational processes to engage a wide-range of interprofessional services in the care of this group. There was no consistent reporting of outcomes or processes in place to measure the impact of interprofessional services for this population.

**Conclusions:**

This study provides important insights into the current state of interprofessional primary care for adults with IDD in Ontario and highlight a critical need for further work in the field to develop organizational structures and processes to engage in team-based care and demonstrate the value of the approach for this population.

**Supplementary Information:**

The online version contains supplementary material available at 10.1186/s12875-021-01502-z.

## Background

Increasing access to interprofessional primary care has been a significant focus of health system transformation in Canada in order to improve the health of all Canadians [1–3]. Interprofessional primary care (also referred to as team-based care) is the provision of a wide range of health services by a team of health providers committed to delivering comprehensive, coordinated, high-quality primary care [4, 5]. There is not one approach to interprofessional primary care provision within or across provinces in Canada and a range of team-based models of care exist including: Family Medicine Groups in Quebec, Primary Care Networks in Alberta, and My Health Teams in Manitoba [6, 7]. 

In Ontario it is estimated that 25–30% of the population currently access primary care through one of the four models of team-based primary care available [1, 8]. Family Health Teams (FHTs) serve approximately 20% of the population and are the most prevalent team-based approach [1, 9]. FHTs are typically oriented to meet the unique needs of their community, and differ in size, organization, team composition, governance and range of programs offered [10]. FHTs are the model most aligned with the core principles of the College of Family Physicians of Canada- *Patient’s Medical Home*, a comprehensive team-based approach to primary care with family physician leadership [11]. Community Health Centres (CHCs) are the second most prevalent team-based model of care in Ontario serving approximately 4% of the population [12]. CHCs were among the first team-based primary care models in Canada, and are characterized by: community governance; a focus on population needs and social determinants of health; an expanded scope of health promotion; outreach and community development services; and salaried interprofessional teams [9, 12]. A limited number of Nurse-Practitioner-Led Clinics and Aboriginal Health Access Centres are available. Nurse-Practitioner-Led Clinics are characterized by: nurse practitioner leadership at all levels of the organization, nurse practitioners and registered nurses working to full scope to provide comprehensive and collaborative primary care, increased access to a range of interprofessional programs and services, engaging patients as full partners in their care plan and a non-profit governing board [13]. Aboriginal Health Access Centres have been in place in Ontario since 1995 and are characterized by: programs that are led by the aboriginal community, services that focus on traditional healing, primary care and cultural programs, as well as health promotion, and community development [14].

Regardless of approach, all interprofessional primary care teams share common aims to optimize patient and population health outcomes, improve quality of care, increase capacity and access to care and support a sustainable health system [15]. Considerable effort has been made to measure the value and impact of interprofessional primary care teams in Canada; however, there is still much to be learned [9, 16, 17]. It is well recognized that the delivery of interprofessional primary care is complex, and influenced by several factors including health policy, the needs of patients and health providers, as well as organizational attributes. Organizational attributes are “the policies, resources, organization and financial arrangements influencing the accessibility, availability and acceptability of medical care services” ([18] p.574). Greater attention to organizational attributes is considered an important element in understanding and predicting health service use [19] as it can impact team performance and the ability to enact coordinated, collaborative care as envisioned [20].

The existence of different team-based models of care in Canada provides both a challenge, as well as an opportunity to better understand how teams function to support and improve the health of Canadians. Fortunately, there have been efforts to understand the organizational attributes of high functioning teams [21]. Gocan, Laplante & Woodend [10] provide a comprehensive review of key organizational attributes for Ontario’s FHTs that include: adequate funding, remuneration and human resources, electronic medical record integration, clarity of vision, effective leadership, clearly defined roles and scope, a patient-centered approach, processes to ensure the patient is seen by ‘right’ professional, communication, shared time and co-location. Beaulieu and colleagues’ [22] cross sectional study of 37 primary care practices in Quebec identified similar characteristics strongly associated with quality of care including: physician remuneration method, extent of sharing administrative resources, presence of interprofessional health providers, and additionally, mechanisms for evaluating competency and organizational accessibility. Russell, Dahrouge, Tuna, Hogg, Geneau and Gebremichael [23] also used a cross-sectional, mixed methods approach to study 137 practices in Ontario and found that practice location (> 10 km to nearest hospital), practice size, the diversity of health providers and practice maturity were significant attributes for comprehensive care. These attributes partially explained the better performance of CHCs in regard to comprehensiveness than in other primary care models such as physician-based Fee-For-Service or Family Health Groups [23].

Although organizational attributes that facilitate high quality interprofessional primary care have been identified, it remains unclear what attributes specifically support care for complex and vulnerable populations. Adults with intellectual and developmental disabilities (IDD) are one such population. It is estimated that between 1–2% of the population has an IDD [24, 25]; IDD referring here to a broad range of developmental conditions that originate before age 18 and involve significant lifelong limitations in cognitive and/or adaptive functioning [26]. Adults with IDD live with complex physical and mental health conditions [27], use health services differently than the general population and face unique challenges with access to health services [28–30].

Providing accessible and appropriate primary care that targets health promotion and chronic disease prevention/management has been identified as critical to improve the health of this group and reduce overall health system costs [28, 31, 32]. Greater access to interprofessional primary care teams that offer a range of health services and health providers has been recommended as an intervention that could address the unique health needs of adults with IDD [33]. However, to date, there is limited evidence regarding the degree to which interprofessional primary care teams are meeting the needs of adults with IDD [34], and how these models of care impact health outcomes and health service utilization [35]. A better understanding of the current provision of interprofessional primary care within and across models of care is needed to identify and explore the influence of organizational attributes in the provision of high quality, interprofessional primary care for adults with IDD [29, 34].

The objective of this paper is to describe organizational attributes related to interprofessional primary care for adults with IDD within and across team-based approaches in Ontario, Canada. This study is the first to describe organizational attributes that facilitate interprofessional primary care for this population and provides the groundwork for understanding how attributes influence care provision and outcomes for individuals with IDD and other complex and vulnerable populations.

## Methods

### Research design

A descriptive, multiple case study design was used to explore the primary question: how is interprofessional primary care provided to adults with IDD within and across team-based models of care in Ontario? Case studies are appropriate for questions that aim to understand: 1) a contemporary phenomenon from multiple perspectives within a real-world context; and 2) phenomena that are likely to involve important contextual conditions over which the researcher has little or no control [36]. The case was defined as ‘*interprofessional primary care for adults with IDD’* and the descriptive case study design allowed for the opportunity to gather a detailed description of this approach through the consideration of multiple data sources and perspectives (e.g. health providers, staff, caregivers and patients) [36]. Use of a multiple case study approach allowed for examination of organizational attributes within and across available models of team-based primary care.

The study was completed over a 21-month period from June 2017 to February 2019.

### Case and participant recruitment

Health services vary across Ontario due to differences in both demographic profiles and regional governance structures [37]; therefore, examining health regionally was determined to be an important contextual condition [35]. The region chosen for the study had the highest prevalence and age adjusted prevalence estimates for adults with IDD in the province at 1.44 and 1.51 percent respectively [38], and this represented a unique opportunity to explore how interprofessional primary care teams were supporting these individuals. In 2009/10, it was estimated that 4,610 adults with IDD (between the ages of 18 and 64 years) lived in the region [29] and the higher prevalence of this population compared to other regions is thought to be related to the multiple institutions for adults with IDD care that were historically located in this area [39].

All interprofessional primary care teams located in the region were identified and organized according to model of care and geography. In total, 15 FHTs, five CHCs and two Nurse-Practitioner-Led Clinics were identified as potential practices. To meet eligibility criteria, all potential practices needed to provide interprofessional primary care, as well as confer care to adults with IDD by the team. No incentives were provided to practices to participate in this study. For each case, the primary author sent invitations to participate in the study to key informants (e.g., lead administrators) by email and followed up by phone. A total of ten practices were initially contacted; the first three FHTs that were invited agreed to participate in the study. The primary author approached all five CHCs and both Nurse-practitioner Led Clinics; three CHCs, and the two Nurse-Practitioner-Led Clinics declined to participate in the study. A final convenience sample of two CHCs, two FHTs and one academic FHT were selected.

Perspectives were sought from a wide range of participants within each team including administrators, health providers, and patients. Recruitment occurred through purposeful sampling strategies including initial criterion sampling to recruit individuals in leadership roles, and subsequent snowball sampling to identify health providers, staff and patients [40]. Recruitment of participants in each case remained open until data saturation was achieved.

### Data collection

Multiple methods were used to generate data including: a survey, document review, an electronic medical record (EMR) practice report, and interviews. The ability to collect data from multiple sources is considered one of the strengths of case study research and allowed for both a comprehensive understanding of the phenomenon of interest, as well as data triangulation [36]. A brief overview of all the methods used is described:

An adapted version of the *Measuring Organizational Attributes of Primary Health Care Survey* [41] was completed by a key informant (e.g. executive director, clinic manager) in each case. The survey provided information on organizational vision, organizational resources, organizational structures, service provision, clinical practice and organizational context. The survey is one of three *Primary Health Care Practice-Based Surveys* developed by the Canadian Institute of Health Information in collaboration with Canadian primary care researchers and survey experts [42]. The surveys are available in both English and French and can be used separately or together to provide a comprehensive understanding of primary care services [42].

A document review was completed by the primary author to identify organizational attributes associated with the provision of patient-centered care and interprofessional primary care for vulnerable populations. The resources included in the review were comprised of publicly available documents including website information, organization policies, strategic and quality improvement plans. An extraction tool was developed to ensure consistency in data collection between cases and focused on organizational attributes such as accessibility, availability and acceptability [18].

An EMR practice report was completed to identify adult patients with IDD currently receiving care from the interprofessional primary care team. As there is no agreed upon approach to the identification of the IDD population within interprofessional primary care practices in Canada the primary author developed an EMR search strategy to assist each team in identifying this population. The search strategy was informed by the local academic FHTs algorithm to identify individuals with IDD in their practice. It is acknowledged the search strategy was not exhaustive however, it did include a list of fifteen common conditions associated with IDD. The conditions included in the EMR search were: (mild, moderate, severe, profound) Mental Retardation, Down Syndrome, Fragile X Syndrome, Prader-Willi Syndrome, Smith-Magenis Syndrome, 22q11.2 Deletion Syndrome, Autism Spectrum Disorder, Asperger’s Syndrome, Rett Sydrome, Williams Syndrome and Cerebral Palsy.

Interviews were completed with administrators, health providers and patients to describe interprofessional primary care for adults with IDD. The semi-structured interview guides were informed by Aday and Andersen’s [43] *Behavioral Model of Access* and questions focused broadly on attributes such as: characteristics of health delivery systems, utilization of health services and service user satisfaction. The interview guides were pilot tested by three clinician-researchers and a research assistant, as well as an individual with IDD. Feedback from the pilot testing was used to refine the questions. Interviews were conducted by the primary author in-person or over the phone based on the preference of the participants. All interviews were audio-recorded on an encrypted external device and uploaded to a University approved secure platform for transcription.

### Data analysis

A-priori propositions were developed to guide the analysis and describe characteristics of interprofessional primary care for adults with IDD within the local health service context. Propositions were developed based on a review of the literature related the population of interest, access to interprofessional primary care for this group, and theories on health service access and utilization. The propositions were:

1. Interprofessional primary care for adults with IDD is supported by organizational attributes (e.g. co-location, shared electronic medical record, access to an interprofessional team).
2. Adults with IDD are supported within primary care teams; however, the extent to which they are identified as a population of interest by the organization will vary between teams.
3. There are limited processes in place to formally engage a range of health providers in interprofessional primary care for adults with IDD.

A within-case analysis was completed for each case and all data sources were initially collected, analyzed independently. Descriptive statistics were used to analyze the survey results. Frequencies were used to provide descriptive summaries of the site, team composition, resources and overarching −–−–organizational context in which interprofessional primary care was delivered. Tables were also used to present results from the cross-case analysis. A directed content analysis was used to analyze available organizational documents in the document review [44]. The primary investigator reviewed all documents and coded all references to organizational attributes such as accessibility, availability and acceptability [18]. Descriptive statistics were used to present results of the EMR practice reports. Prevalence of the population served by the interprofessional primary care team was identified by calculating the number of adults with IDD currently supported compared to the total number of patients enrolled in the practice.

All interviews were initially transcribed and anonymized by a professional transcription service. Interviews were uploaded to MAXQDA [45], a qualitative data management and analysis program, and each case was saved as a separate project. Thematic analysis [46] was used and investigators followed the main steps in this approach including familiarizing one’s self with the data, generating initial codes through line by line coding, searching for themes, reviewing themes, defining and naming themes [46]. Two authors (NB, CD) independently coded the first three transcripts and discussed the development of initial codes. Revisions to the coding frame and categories were reviewed and changes made as needed during the individual case analysis. The coding frame was exported and applied to the analysis of the four remaining case studies, and additional codes were added as unique concepts were identified.

Pattern matching was considered the main case study analytic approach [36, 47]. Pattern-matching techniques were specifically used to identify and compare patterns in the data against the hypothesized propositions [36]. Within each case a pattern matching process was completed to synthesize and converge the results obtained from the analysis of multiple methods (Supplementary Figure 3) [36, 47]. A cross-case synthesis was then completed to compare and contrast organizational attributes of interprofessional primary care for adults with IDD within and across models of team-based care. Tables and matrices were used to display results. In the final phase, a theoretical and conceptual lens was used to assist in describing the organizational attributes of interprofessional primary care for adults with IDD based on literature regarding primary care organizational attributes and health service access for vulnerable populations (Supplementary Figure 4 ).

### Rigour

A number of case study strategies were used to ensure rigour [36]. The primary author maintained a reflexive journal and audit trail as well as, completed field notes and memos. A database and case study protocol were developed and applied consistently across cases to ensure replicability. Dependability was increased through the use of standard data collection forms (e.g. EMR search strategy, document review) and published survey [41]. Every attempt was made to approach each case using the same protocol; any minor variances that occurred were documented and discussed with the primary author’s supervisor. Member checking was completed, and transcripts were reviewed by interested participants (N = 2). Initial results were also presented during team meetings at two of the five cases. Finally, to assist with transferability, case summaries were completed to provide thick descriptions of interprofessional primary care for adults with IDD within a real-world context.

## Results

General site profiles were compiled for each team (Table 1). A summary of participants (N = 43) is also provided by site and professional role in Supplementary Table 1 as well as a summary of selected organizational attributes is provided in Supplementary Table 2 .

**Table 1:** Site Profiles

Characteristics	Case 1	Case 2	Case 3	Case 4	Case 5
Primary Care Model	FHT	FHT	CHC	FHT	CHC
Governance	Physician-Led	Physician-Led	Community-Led	Physician-Led	Community-Led
Physician Remuneration^a^	Blended	Blended^b^	Salary	Blended	Salary
Practice Locale^c^	Small Town	City	Rural (2 Sites)	Small Town	Small Town + Rural Sites
Operation (Years.)	10 +	10 +	10 +	10 +	10 +
Practice Size (Patients)	13 881	12 682	5589	20,260	5000
Team Composition	**(FTE)** 12 GP (10.0) 3 NP (2.6) 1 Pharm (0.6) 1 RN Sys Nav. (1.0) 3 RN (3.4) 1 RD (0.6) 1 SW (1.0)	**(FTE)** 23 GP (21.2) 1 NP (1.0) 1 Pharm (1.0) 7 RN (3.5) 1RD (1.0) 2 SW (2.0) 17 RPN (12.0)	**(FTE)** 4 GP (3.8) 3 NP (3.0) 1 Pharm (1.0) 1 RN Sys-Nav. (1.0) 4 RN (4.0) 1 RD (1.0) 2 SW (1.1) 1 RT (0.4) 1 Geri-Psych (< 0.1)	**(FTE)** 23 GP (23) 4 NP (4.0) 1 Pharm (0.8) 7 RN (7.0) 2 RD (0.9) 4 SW (3.0) 1 Psych (< 0.1)	**(FTE)** 3 GP (3.0) 6 NP (4.8) 1 Pharm (1.0) 1 RN (1.0) 1 RD (1.0) 1 SW (1.0) 2 RT (1.5) 1 Geri-Psych (< 0.1)

Key: Full Time Equivalent [FTE]; Geriatric Psychiatrist [Geri-Psych]; Nurse Practitioner [NP]; Pharm [Pharmacist]; Physician [GP]; Psychiatrist [Psych]; Registered Dietitian [RD]; Registered Nurse [RN]; Registered Practical Nurse [RPN]; Respiratory Therapist [RT]; Social Work [SW]; Systems Navigator [Sys Nav.] ^a^ Physician Remuneration – Blended model provides mix of payment models including capitation and fee-for service payments; Salary – GPs provided annual salary (which could include hourly rate, sessional payment). ^b^ Case 2 Blended model includes funding for GPs provided by academic institution. ^c^Practice Locale descriptors (e.g. rural, small town, city) are included as provided in Measuring Organizational Attributes of PHC Survey (CIHI, 2017); no further detail available

### Site profiles

#### Case one

Case one is a FHT with one main clinic site for health providers and four additional practice sites. The organization was a part of the initial cohort of FHTs in Ontario and has been providing primary care in the community since 2006. The FHT currently has 13,881 patients enrolled with 12 physicians and three nurse practitioners. The organization is led by an executive director who is responsible to a board of directors composed of physicians affiliated with the organization.

and two community members. Physicians are required to pay their own overhead and have their own office suites, nurses and administrative staff. Health providers are co-located on the main clinic site, although they may work part-time at the other sites. All health providers and staff associated with the FHT share an EMR.

#### Case two

Case two is an academic FHT with two main clinic sites. Academic FHTs are interprofessional primary care teams that deliver care “in an environment in which family medicine residents, medical students, and other health professional learners are trained” ([48]^.^p.e25). In this case, the academic FHT is associated with a university’s school of medicine and there is a strong focus on family medicine resident training. The academic FHT has been providing primary care in the community since 2006 and has 12,682 patients enrolled with 23 physicians. The FHT has a physician advisory board. In case two, physicians are not required to pay overhead for space, materials or staff. Health providers are located at either of the two main clinic sites which are situated within walking distance of each other. All health providers and staff associated with the FHT share an EMR.

#### Case three

Case three is a rural^1^ CHC with two main clinic sites. The CHC has been serving the community for over 30 years and currently has 5,589 patients rostered with four physicians and three nurse practitioners. The organization is led by an executive director and community board of directors. All physicians receive a salary and are not required to pay overhead. All staff are co-located and share resources, as well as an EMR. Guided by the Alliance for Healthier Communities guiding framework [49], this organization focuses on access to health for everyone, including an explicit reference to vulnerable residents (e.g., seniors, families on low income, people with disabilities and people who are isolated). Furthermore, the organization also partners with local health and social service agencies to co-locate and/or collaborate to bring services to local citizens.

#### Case four

Case four is a FHT with two main clinic sites and two additional physician offices. The organization was part of the initial cohort of FHTs in Ontario and has been part of the community since 2006. The FHT currently has 20,260 patients enrolled with 23 physicians. The organization is led by an executive director and a community board of directors who are predominantly physicians. Each physician is required to pay overhead and has their own office suites with separate nurses and administrative staff. All health providers and staff associated with the FHT share an EMR and many of the health providers are co-located in offices in the two main clinic sites. Select programs are run in collaboration with the local hospital and community health and social service agencies (i.e., palliative care and cardiac rehabilitation program).

#### Case five

Case five is a CHC with two main clinic sites. The CHC has been serving the community for over 10 years and currently has 5,000 patients enrolled with three physicians and six (4.8 full time equivalent) nurse practitioners. The organization is led by an executive director and a community board of directors. All staff are co-located and share resources, as well as an EMR. The CHC partners with local health and social service agencies to co-locate and/or collaborate to bring services to local citizens and is guided by the CHC’s practice framework [49].

### Organizational attributes and interprofessional primary care for adults with IDD

The cross-case analysis identified three major themes related to organizational attributes and the provision of interprofessional primary care for adults with IDD; the themes were: 1) enabling attributes (organization and resources); 2) being seen –gaining entry through identification; 3) targeted programs for the care of adults with IDD.

### Enabling attributes (organization and resources)

The first theme addresses the initial study proposition. There were seven enabling organizational attributes related to interprofessional primary care of adults with IDD including: having a vulnerable population and disability orientation; supportive leadership; an interprofessional team; shared EMR; co-location; having time, and scheduling flexibility.

#### Having a vulnerable population and disability orientation

Both CHCs in the study were well-established in their respective communities, were informed by principles of equity and attention to social determinants, and had well-developed policies around accessibility (e.g., pledge and plans). There was consistent messaging across documents in both cases and their accessibility in clinic and on the website resulted in increased confidence that these were organizational priorities.

Although also well-established in their communities FHTs were newer teams as compared to the CHCs (i.e., 15 years versus 30 + years). Physical accessibility was available; however, additional accessibility features and associated policies were less developed and also not consistently featured across the participating FHTs compared to the CHCs. With respect to having a vulnerable population orientation, the academic FHT (Case two) noted in their vision of care a commitment to serving individuals with IDD. The remaining two FHTs had no official document identifying service processes to care for vulnerable populations. As one FHT administrator noted:

So, we’ve never really looked at a sub-population of adults with intellectual disability, right? The same way that, say, the CHC would be, because their focus is on social determinants and marginalized populations. 1_Admin_1

#### Leadership

Supportive organizational leadership was identified as an enabling element in interprofessional primary care provision for adults with IDD. In two of the five cases, leadership supported individuals with IDD to be identified as a population of interest for the practice. As an administrator described:

So, my, our ED (Executive Director), who’s now retired, um, very progressive and very community-based man who, who capitalized on this idea. [The IDD community nurse] was seen as a credible resource from within the community. 5_Admin_1

#### An interprofessional team

From a resource perspective, having an interprofessional primary care team with varied expertise was explicitly acknowledged as valuable across all cases (CHCs and FHTs). As one physician noted, “it’s all really helpful because those people have a depth of knowledge in their areas that I can’t, you know, replicate” 3_HCP_1. Having access to a range of health providers to support the care of these patients was recognized:

Because honestly, given the fact that everybody’s time is limited and my own personal philosophy in terms of a collaborative care environment is: if I’m not the best person for doing this particular function, I shouldn’t be trying to do it; that person should be. 1_HCP_1

I recognize, having been in sole family practice, that I wasn’t able to provide the, the level of care doing that, that I am now with the extra people on the team. 3_HCP_1

#### Shared EMR

A shared EMR was identified as a critical factor to facilitating communication among health providers on the team. As one health provider noted:

Within our health team, communication is quite good, because we’re all on the same medical record. And so, you know, if the dietitian sees someone, she sends me a message and then I can see her note, and she doesn’t hesitate to come by my office…2_HCP_2

Shared records were also considered a support for the provision of interprofessional primary care for this population, one health provider noted “within the team, at least records can be shared, and that’s one of the advantages of here is that we do share records” 4_HCP_1. The benefit of the shared records was described as “I can read the doctor’s reports and the doctors, of course, can read mine” 4_HCP_2 which facilitates more coordinated care.

#### Co-location

Co-location of health providers was identified as an important condition of interprofessional primary care provision across all cases. As one health provider described “I sit on one side of the building, then I just walk down the hall. I’ll say, ‘Yes, we need to discuss, discuss these cases, okay.’ And so we close the door and then we’ll meet for whatever time we can about this” 3_HCP_2. One difference noted, was that additional opportunities to coordinate care with other health and community service agencies were more readily available for the two CHCs than the FHT teams as the CHCs both co-located with other community service agencies. This was seen as a benefit as the co-location facilitated communication, for example, as one health provider noted: “We have one of them that, ah, [a service coordinator], she comes out a couple times a month, so I sit down and chat with her about all our [shared] clients that, ah, might be struggling a bit” 3_HCP_3. This ability to facilitate more coordinated care is of particular relevance to adults with IDD as they are known to require both health and social services.

#### Having time

Having the time to care for adults with IDD with additional needs and complexity was identified as critical. As one CHC physician noted:

The most important thing when you take care of these -- you know, when you’re part of that team, you need time. And the practice has to give you the time to give the patients the time that they need. Because they have special needs and special needs translates into extra time. 5_HCP_2

This is reflected in other statements regarding a health provider’s ability to decide how long to support someone “as long as it takes”3_HCP_3. However, remuneration impacts time use and finding additional time was difficult for physicians associated with models (e.g. FHTs) that rely on blended remuneration options (e.g. capitation and fee for service payments) versus salaries as provided by CHCs or alternative funding models as seen by the academic FHT. As one FHT health provider noted: “So, that’s part of the other problem, I think is that, um, the way Health Care’s set up, particularly it’s Fee for Service—It just isn’t set up for, for that kind of time to spend together” 4_HCP_1. This theme of limited time also resonated with health providers in the FHT model, especially in regard to social work services as one health provider mentioned: “our role here is supposed to be short-term. The maximum of six to eight sessions, but when you’re dealing with somebody that has that many challenges, um, it’s not always that easy” 1_HCP_3.

#### Scheduling flexibility

Further to having time, a certain degree of flexibility and professional discretion was reported in regard to scheduling for interprofessional primary care provision in both CHCs and FHTs. Flexibility on how and where to conduct their visits (i.e. home, community or clinic) was identified as enabling, “Yeah, we have a choice…Especially if – Well, especially if they don’t have access to transportation, or it’s difficult for them to get here” 3_HCP_3. Having scheduling flexibility to accommodate patients was also seen to be a facilitator to care, “I will adjust my schedule when I know that there’s somebody out there that needs a hand, right*?*” 4_HCP_3. The health providers noted that this is especially pertinent in the care of adults with IDD who may have challenges with access to transportation, availability of care givers and/or when attending medical appointments causes additional stress.

### Being seen- gaining entry through identification

**Fig. 1:**
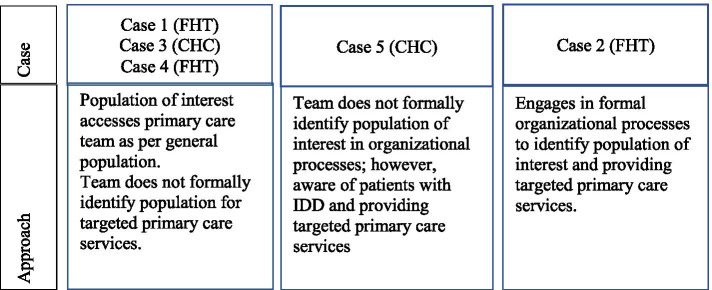
Approaches to identification of adults with IDD across cases

The next theme directly relates to the second proposition and the extent that adults with IDD are identified at an organizational level. Results supported the proposition that while adults with IDD were found to be supported within all interprofessional primary care teams (Table 2); the extent and methods of formally identifying this population at an organization level were not consistent within or across cases. The cases could be described as falling within three main approaches in regard to identification of this population (Fig. 1). The type of model of care did not appear to influence the decision to formally identify adults with IDD as a population of interest.

**Table 2:** Number of adults with IDD in interprofessional primary care teams across cases

Primary Care Team-IDD Population Orientation	Case 1 FHT	Case 2 FHT	Case 3 CHC	Case 4 FHT	Case 5 CHC
Number of Patients with IDD Identified (%)	91 (0.66)	219 (1.73)	14 (0.25)	223 (1.10)	82 (1.6)
IDD Program of Care	No	Yes	No	No	Yes

The academic FHT (Case two) was the only organization that had engaged in a service process to formally identify this population within their EMR. Strong organizational leadership, physician advocacy, clinical interest and alignment with the organization’s strategic pillars were contributing factors to the establishment of an identification process. As one of the administrators noted:

we do have fairly formal processes and, because we have been looking at this population, [we] have such an interest, both in our research and education of physician residents, and just clinical outcomes. So, all three of our, the pillars of our organization are interested in us identifying and re-calling and providing preventative care for this population. 2_Admin_2

A second team (Case five) had an IDD community nurse who was personally aware of the individuals with IDD in the practice and could identify individuals through a targeted EMR search of the team’s IDD clinic, where most individuals with IDD were rostered to one physician. No other team had organizational processes in place to identify this population within their practice. These teams cited common challenges including a lack of agreement of use of diagnostic codes and a lack of appropriate assessments to support a formal diagnosis. Providers noted burden and frustration with limited access to appropriate assessment services especially in adulthood.

Although IDD was recognized by the health providers and patients, one CHC physician noted: *“*the big problem is: if people weren’t identified before age 18, forget it. Forget it, you know. Nothing’s going to happen for them. And I have seen that*”* 3_HCP_3. A nurse system navigator supported that sentiment citing long wait lists for assessment services: *“*My frustration is the waiting, waiting time and (laughs) it’s like, ‘He needs it yesterday.’ And he’s still on the waiting list, so that’s my biggest frustration” 3_HCP_2. This was further reiterated by a physician in a FHT:

I’m referring specifically to things like a clinical psychologist doing appropriate assessments. I’ve always found that there’s a huge barrier to getting that type of work done. Now, in a few cases that I can think of off the top of my head, there’s probably been via efforts, say, through Social Services and stuff to engage a clinical psychologist to get somebody assessed. But there’s fair bit of work to get that organized. And there can be some significant delays getting those things done. 1_HCP_7

One final challenge was a lack of recognition of benefit to health providers to formally identify this group as a population of interest. As one team administrator noted: *“*So, if you find those patients, so what does that mean for you? And now that you have that knowledge, what do you do with it*?”* 1_Admin_1*.* She went on to further state: *“*I think the IDD piece is relevant in terms of how we deliver the programs, not whether they’re accessing the programs” 1_Admin_1.

### Targeted programs of care for adults with IDD

This final theme focuses on two targeted approaches to the provision of interprofessional primary care for adults with IDD. A cross-case comparison is provided for these two programs in order to describe key attributes.

#### Cross-case comparison

Two of the cases, one CHC and one FHT, had targeted programs for the care for adults with IDD (Table 3). These programs both benefited from enabling organizational attributes such as co-location and a shared EMR to facilitate communication. They also share many program-level features such as the same core professionals involved (i.e., physicians, nursing), the types of services offered, opportunities for collaboration and coordination, as well as promote similar processes around accessibility/accommodation. In both cases, the engagement of a range of health providers (beyond nursing) was minimal and these health providers were referred to on an as needed basis. Despite these similarities, the programs were found to be relatively distinct in how they were funded, and consequently how they were developed and delivered within their respective organizations. One of the primary organizational differences is the degree to which these programs were established within formal service processes. In Case two, the program has established care processes (operationally defined at the organizational level) and the program is incorporated into quality improvement initiatives. In Case five, the program of care has established care processes; however, they are mainly unrecognized at the organizational level (e.g., they are individual practice processes enacted by those most responsible for care) and the program is also not formally evaluated. Case descriptions are provided for both programs as supplementary files.

**Table 3:** Cross-Case Analysis of Targeted Programs of Care for Adults with IDD

**IDD Program of Care**	Case 2 FHT	Case 5 CHC
**Main Purpose of Program**	Provide Health Check	Care Coordination
**Age of Program**	5 Years (est. 2014)	12 Years (est. 2007)
**Organizational Support**	• Yes	• Yes
**Core Health Providers-** **Staff Currently Involved** ***(Resources- Health Personnel)***	• Physicians/ Residents • Nurses • Clinic Clerk (scheduling)	• Physician • IDD Community Nurse • Clinic Clerk (scheduling)
**Provider Remuneration** ***(Resources – capital)***	Physicians: Blended model Residents Nurses, Clinic Clerk: Salary	All providers: salaried Community IDD nurse salary funded by Ministry of Children, Community and Social Services
**Services Offered** ***(Resources – health personnel, equipment, materials)***	• Care Coordination • Health Check • Usual Primary Care • System Navigation • Advocacy	• Care Coordination • Usual Primary Care • Health Teaching • System Navigation • Advocacy
**Access to Program (*Organization- Entry*)**	Rostered with physician associated with case 2. Patients may have suspected or known IDD	Open access to nurse if known or suspected IDD; typically roster with physician associated with IDD clinic if possible
**Availability (*Organization- Entry*)**	Monday to Friday. On call services available after hours	Monday to Friday. Nurse offers emergency cell on-call services after hours
**Accessibility-Accommodation** **(*Organization-structures)***	Visits offered in clinic or group home/residence as required	Visits offered in clinic or group home/residence as required
**Opportunities for Collaboration** **(*Organization-structures)***	Yes, although not typical. Main providers physicians; however, can refer to all health providers as needed	Yes, collaboration between physician and nurse; however, can refer to all health providers as needed
**Opportunities for Coordination** **(*Organization-structures)***	• With Developmental Services Agency staff and specialist IDD health providers	• With Developmental Services Agency Staff
**Program Evaluated** ***(Organizational -structures)***	Yes, evaluation identified in quality improvement plan	Not at this time

## Discussion

This is the first study to offer insights into the organizational attributes that support interprofessional primary care for adults with IDD in Ontario. As Glazier et al. [9] notes, context is an important consideration in primary care performance and, the co-existence of different team-based models of care within the same geo-political environment provided an ideal opportunity to describe similarities and differences in the provision of interprofessional primary care for this group [50]. Both FHTs and CHCs have been identified as ideally suited to meet the needs of adults with IDD given a close connection to their communities and interprofessional approach [51].

With regards to organizational attributes, the results largely reiterate and support what is known about high-functioning interprofessional primary care teams. A common vision, supportive leadership, an interprofessional team that is co-located and access to a shared electronic medical record are well-recognized organizational attributes in successful teams [1, 4, 10]. Harris and colleagues’ study on the effectiveness of interprofessional teams noted similarly that “colocation facilitated getting to know one another, building trust, and establishing new practice patterns. Trust, in turn, made developing shared goals possible” ([52] p.41). In addition to being perceived to improve contact between team members and increase collaboration, co-location offered increased convenience for patients [53]. A shared EMR was also important to facilitate virtual interprofessional communication and shared care of patients. It is important to note here that the presence of organizational attributes alone does not guarantee the engagement of an interprofessional primary care approach; as Harris [52] noted, “effective communication strategies whether face to face or virtual, were recognized as essential if trust, respect and common understanding were to be achieved. Without these, co-location alone did not achieve desired outcomes”. p. 41.

In terms of similarities, all of the teams were identified to have these organizational attributes; however, the degree to which they were optimized within the organizational culture and daily practice varied. All teams identified that they supported vulnerable populations in their practice, including adults with IDD and people with low socio-economic status, refugees and rural patients. With regards to models, the CHCs more so than the FHTs were found to have well established processes around accessibility and a culture of interprofessional primary care for vulnerable populations. This is likely due to the maturity of the model and the long-standing mandate to address social determinants of health within CHCs [12, 23]. Given the addition of social accountability in the updated principles of the College of Family Physicians of Canada’s *Patient’s Medical Home* it is anticipated that FHTs will need to develop more organizational recognition and subsequent policy and service processes around social determinants of health to support their more vulnerable patients. The study supported the sentiment of Beaulieu and colleagues [22] who argued that high-quality care can be achieved by practices with different organizational models and that “how the work is organized may be as important, if not more than what the model is called” p. E590.

Importantly, the results also highlight the diversity of interprofessional primary care teams. No one team was found to be exactly the same in regard to organizational attributes (e.g. team composition, available programs) and this was identified within and across models of care. Although it could be argued that this is a positive finding as it allows for teams to reflect their individual community needs, the lack of consistency in approach across teams makes it challenging to navigate and measure the impact of the approach at a broader systems level.

One of the critical findings that influenced interprofessional primary care for this group was the degree to which this population was identified within the interprofessional primary care team. The type of model (CHC or FHT) did not appear to matter in this regard and individuals with IDD remain largely unidentified at an organization level. At the time of data collection, four sites (two FHTs, two CHCs) had no formal organizational service processes established to identify individuals with IDD, although in one CHC all individuals were known to the IDD community nurse. This finding is not surprising given that individuals with IDD made up a small percentage of the total practice rosters of these teams. However, in light of the fact that adults with IDD are known high-cost health system users [31] and use the emergency department more often than individuals without IDD [28] it is concerning that they are not identified for more targeted monitoring at the primary care level. Acknowledging the potential challenges in identifying individuals with IDD at a primary care practice level (e.g., broad spectrum of conditions, lack of supporting documentation for diagnosis), Ontario’s Health Care Access and Research on Developmental Disabilities [HCARDD] program has provided publicly available practice resources to support organizations in establishing service processes to identify this population with EMRs. Now it is a matter of uptake. As noted in this study, supportive leadership and practice champions are needed to establish initiatives such as the identification of adults with IDD into regular programming and quality improvement.

Greater identification of individuals with IDD at an organization level has implications more broadly regarding health services utilization. Health systems continually evolve and there is an ongoing possibility that vulnerable populations such as adults with IDD are lost within these health system transformations. There is a need to be vigilant about the impact of reform initiatives on populations such as adults with IDD who experience challenges with access to appropriate health service and poor health outcomes [50]. To ensure seamless transition across health services, it will be important for interprofessional primary care teams to focus on proactively identifying and following individuals with IDD, especially those among this group that require the coordination of many health services and who are at increased risk of high or inappropriate health service use [28].

Even when individuals with IDD are known in the organization, there is still further work needed to engage in an interprofessional team approach. Results indicate that physicians and nurses continue to be the primary health providers involved in care and there is limited engagement of other interprofessional services in the care of adults with IDD. In the Health Check program, the physician acts as the main point of contact and coordinator of care. This program does not require an interprofessional team; however, does increase early detection of conditions and need for further monitoring which has the potential to engage other members of the team [54]. Although in support of a team approach, the Health Check program at this time relies largely on the physician/resident. In regard to the engagement of other health providers, challenges knowing who is needed, resources and scheduling were identified as reasons for the lack of formal engagement of interprofessional services [55]. In the CHC, the IDD community nurse is the main point of contact with health system and acts largely in the care-coordinator and system-navigator capacity. The nurse works in close collaboration with the physician to coordinate care for this group and engage other health providers as needed. While this model has not been evaluated within the practice it has similar processes to nursing roles in other jurisdictions in the United States [56].

*Primary Care of Adults with IDD 2018 Canadian Consensus Guidelines* recognize there are roles for a broad range of interprofessional services specifically around health promotion, chronic disease prevention and management [33]. However, there is currently little guidance on HOW to enact this approach. Moving forward, it will be important to develop processes to engage interprofessional primary care teams and all health providers in the care of adults with IDD to reduce potential inequities in health service use and improve health outcomes. Future work to identify the scope of interprofessional primary care services required by this population would assist in targeted engagement initiatives. Increasing the health system capacity to collect interprofessional health services data in interprofessional primary care teams is another method to facilitate a greater understanding of the current scope of interprofessional services for this population.

### Limitations

The research questions and study design aimed to describe interprofessional primary care for adults with IDD in one region of Ontario and specifically the organizational attributes related to this approach. Further research would be of value to evaluate the impact of interprofessional primary care, (beyond just access to a team-based model) on health status and health service utilization for adults with IDD. In addition, this study is limited by only describing features of two models of interprofessional primary care in Ontario. Further research would be strengthened by exploring how other models of care provide interprofessional primary care for this population, specifically Nurse-Practitioner Led-Clinics which have a practice focus on chronic conditions and collaborative interprofessional practice and Aboriginal Health Access Centres. Finally, the description of the scope of interprofessional services was limited to what was collected from the survey, document review and qualitative interviews. A manual audit of the EMR to identify the extent of involvement of health providers and scope of service for this population and/or access to population level data of health providers in primary care would have provided a more robust understanding.

## Conclusions

Improved understanding of the organizational attributes that support or hinder the delivery of high quality interprofessional primary care is important [6, 34, 57]. This research contributes to our understanding of the organizational attributes that enable interprofessional primary care specifically for adults with IDD, a known complex and vulnerable population. Results confirm that interprofessional primary care provision for this group is facilitated by organizational attributes consistent with high functioning teams. Rather than recommend a specific model of care, these results highlight examples of targeted programs of care for adults with IDD that exist within different interprofessional primary care teams. This knowledge can be used by interprofessional primary care teams to develop approaches to the care of this population within their own practices. In order to make informed organizational policy and planning decisions for vulnerable populations, greater identification of these populations is critical. Identification also assists in providing tailored care approaches that address on-going challenges and inequities that adults with IDD experience in accessing health services. The development of organizational level processes to engage a range of health providers would be of further benefit to optimize the potential of team-based care for this group and is currently lacking in Ontario.

## Supplementary Information

**Additional file 1.**

**Additional file 2.**

**Additional file 3.**

**Additional file 4.**

**Additional file 5.**

## Data Availability

All data generated or analysed during this study are included in this published article [and its supplementary information files].

## Abbreviations

CHC: Community Health Centre
EMR: Electronic Medical Record
FHT: Family Health Team
HCP: Healthcare Provider
IDD: Intellectual and Developmental Disabilities

## Footnotes

1 Rural practices typically support a community of < 30,000 and are greater than 30 min away from a larger community > 30,000, [48].
