# Supplementary material for: Organizational attributes of interprofessional primary care for adults with intellectual and developmental disabilities in ontario, Canada: a multiple case study
Source: BMC Fam Pract. 2021 Jul 22;22:157. doi: 10.1186/s12875-021-01502-z (PMC8299637; doi:10.1186/s12875-021-01502-z)
Supplement: Supplementary file 1 — Additional file 1. [file 12875_2021_1502_MOESM1_ESM.docx]

Table 1: Interview Participants Across Cases

|  | Case 1 FHT | Case 2 FHT | Case 3 CHC | Case 4 FHT | Case 5 CHC |
| --- | --- | --- | --- | --- | --- |
| Participants per case | ED  Physician  (2) NP  (2) RN  SW  RD  DE (RN)  Data Analyst | Manager  (2) Physicians  CSW  Pharmacist  SW  RD  (2) Clinic Clerks  QIDSS  Patient  Caregiver | Director - P&S  Physician  SW  RN- Sys-Nav.  (2) Patients  (2) Caregivers | ED  Physician  NP  SW  Psychiatrist  Patient  DS-SUP | Director - PC  Physician  RN  RD  Patient  DS-SUP |

Key: Community Services Worker [CSW]; Developmental Services Supervisor [DS-SUP]; Diabetic Educator [DE]; Director PC [Primary Care]; Director P&S [Programs & Services]; Executive Director [ED]; Nurse Practitioner [NP]; Quality Information & Data Support Specialist [QIDSS]; Registered Dietitian [RD]; Registered Nurse [RN]; Social Work [SW]; Systems Navigator [RN-Sys-Nav]
