# Supplementary material for: Organizational attributes of interprofessional primary care for adults with intellectual and developmental disabilities in ontario, Canada: a multiple case study
Source: BMC Fam Pract. 2021 Jul 22;22:157. doi: 10.1186/s12875-021-01502-z (PMC8299637; doi:10.1186/s12875-021-01502-z)
Supplement: Supplementary file 2 — Additional file 2. [file 12875_2021_1502_MOESM2_ESM.docx]

| **Characteristics of Health System**  **Organizational Attributes (Selected)** | **Case 1 FHT** | **Case 2 FHT** | **Case 3 CHC** | **Case 4 FHT** | **Case 5 CHC** |
| --- | --- | --- | --- | --- | --- |
| Vulnerable Popl’n  Policy Orientation | No | Yes | Yes | No | Yes |
| Access | Only patients with an active record or registered at the clinic | Only patients with an active record or registered at the clinic. IDD patients - program of care. | Only patients with an active record or registered at the clinic | Only patients with an active record or registered at the clinic | Patients with an active record or registered at the clinic or live in neighborhood served by the clinic for special programs. IDD program of care. |
| Interprofessional Services Provided | Yes | Yes | Yes | Yes | Yes |
| Health Provider  Roles Defined | Yes | Yes | No | Yes | No |
| Publicly available ways to access interprofessional services | Yes-website. 2 Programs Only Healthy Nutrition & Mental Health. | Yes - website. | Yes - website. Typically, through provider, some self-referral. | Yes, website. | Yes, website. 2 programs only, Diabetes and Telemedicine. |
| Co-location | Yes | Yes | Yes | Yes | Yes |
| Shared EMR | Yes | Yes | Yes | Yes | Yes |
| Opportunities for Collaboration | No formal processes;  joint visits possible. | Mental health rounds only. | No formal processes;  case conferences | Yes, interprofessional team programming | Daily huddles, GP/RN clinic; case conferences |
| Programs & Services Offered | Anti-coagulation  Chronic Disease Self-Management  Smoking cessation  Well-Woman  Well-Baby  Diabetes Program  Healthy Nutrition Respiratory Disorders  General Medicine  Hypertension management  Influenza program  Immunization Mental Health &Addictions  (telemedicine) | Anti-coagulation  Diabetes Program  Foot Care  Home Visits  Immunization  Nutrition Counsel.  Minor Procedures  Mental Health Care & Counsel. Medication Safety  Health Links  Coordinated Care  Obstetrics & Prenatal Care  Well-Baby  Well-Child Check  Primary Care- Asthma  Psychiatry  Smoking Cessation  Breast Feeding/  Infant Nutrition  Cancer Screening  Best Health-Best Weight | Health assessments  Periodic health exam  On-site laboratory & ECG services  Immunizations & vaccinations  Pre/post-natal  Early childhood  Nursing foot care & Chiropody  Individual & family Counselling  Social work program  Lung health  Pharmacist  Nutritional counselling  Chronic Disease  Self-Management  Diabetes prevention and screening clinics  Outreach services  telephone health advice  ON SITE Specialty Services: Lung Health, Dental, Geriatric Psychiatry, Arthritis, Adult Day Program, Alzheimer’s Society | Cardiac Rehabilitation  Diabetes Education  Heart Function Clinic  Maternal Infant & Child Program Palliative Care *  Wound Care  Store Forward Project Ontario Telemedicine- Dermatology  Clinical Nutrition  Direct Primary Care-  NP Clinic  Higher Risk Foot Care  Memory Clinic  Pharmacy  Smoking Cessation  Chronic Disease  Self-Management  Telemedicine Network  Coordinated Care  Health Promotion  Lung Health  Mental Health  Visiting Consultants  (Heart Function, Orthopedics,Pediatrics,  Respirology, Urology) | Listed Programs:  Diabetes Services and Telemedicine programs |
| Accessibility-Accommodation | Not explicitly stated in documents and no accessibility statement.  Accessibility features enabled on website. | No documentation available re: accessibility features. No web accessibility features available. | Accessibility Section on website, includes Accessibility Pledge, Accessibility Plan. Website accessibility features available. | Commitment to accessibility noted on website. Website accessibility not enabled. | Accessibility Section on website, includes docs: Accessibility Pledge Accessibility Plan Website accessibility features available. |
