# Supplementary figures and images for: Organizational attributes of interprofessional primary care for adults with intellectual and developmental disabilities in ontario, Canada: a multiple case study
Source: BMC Fam Pract. 2021 Jul 22;22:157. doi: 10.1186/s12875-021-01502-z (PMC8299637; doi:10.1186/s12875-021-01502-z)

Figure 1: Pattern Matching Process for Case Study Analysis


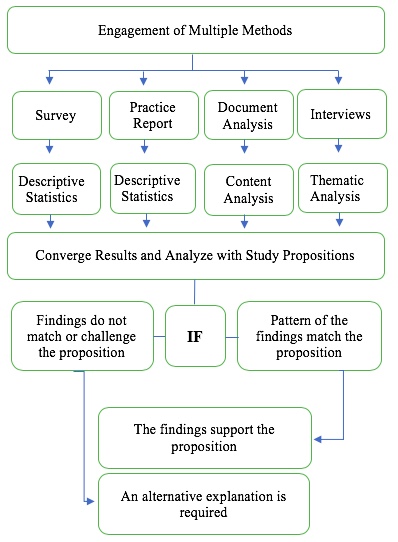

Supplement: Supplementary file 3 — Additional file 3. [file 12875_2021_1502_MOESM3_ESM.docx]

Figure 2: Multiple Case Study Data Collection & Analysis Process


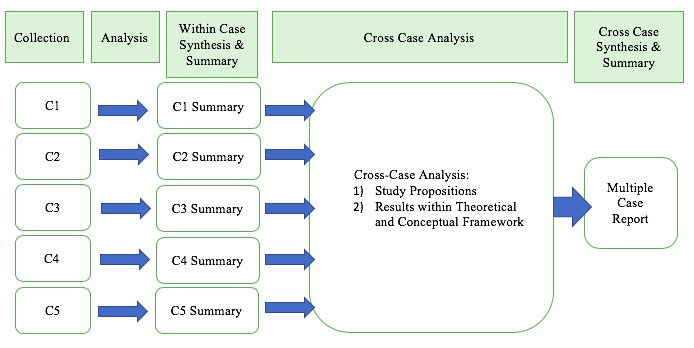

Supplement: Supplementary file 4 — Additional file 4. [file 12875_2021_1502_MOESM4_ESM.docx]
