# Supplementary material for: Organizational attributes of interprofessional primary care for adults with intellectual and developmental disabilities in ontario, Canada: a multiple case study
Source: BMC Fam Pract. 2021 Jul 22;22:157. doi: 10.1186/s12875-021-01502-z (PMC8299637; doi:10.1186/s12875-021-01502-z)
Supplement: Supplementary file 5 — Additional file 5. [file 12875_2021_1502_MOESM5_ESM.docx]

Supplementary Files: Case Summaries IDD Programs of Care

The health check program.

The Health Check program was established in 2014 and involves a full review by a physician every 18 months. All adults with IDD rostered with a physician are eligible for the service and the Health Check is completed in addition to usual primary care. As this is an academic FHT, all physician residents receive training on how to complete a Health Check and complete at least one in their two-year residency. The Health Check follows a standardized form available in the EMR. The first visit takes approximately one hour and is completed either in clinic or, if accommodations are needed, at the patient’s residence.

The development of the Health Check program was supported by multiple stakeholders including the Department of Family Medicine, the physician-lead, identified physician champions, a research team and formal implementation process. The physician-lead for the program was a faculty member with remunerated academic time and this facilitated his work in the development and implementation of the Health Check program. Training was provided by the physician-lead and clinic managers to all staff and health providers. Quality improvement goals for this program have been developed and the focus to date has been to increase the percentage of eligible individuals who receive a Health Check within 18 months.

One of the initial steps in establishing the Health Check program involved a process of identifying all adults with IDD within the practice. An EMR search strategy was developed in consultation with the physician-lead for the program and research team, and the search was completed by the quality information and data support specialist on the team. Once individuals were identified, patient lists were sent to each physician for verification. After the verification process, patients were invited to participate in the program by an assigned clinic clerk by telephone with the help of a standardized invitation script. If patients consented to participate, they were assigned a unique diagnostic code associated with the program (in addition to other relevant diagnostic codes); this decision was made in order to identify the Health Check participants for future program evaluation and quality improvement initiatives.

Initially, all the clinic clerks participated in calling the individuals with IDD identified in their practices to schedule appointments, but difficulties encountered in training and supervising them (including turnover in staff) led the team to assign just one clinic clerk to call everyone. The clinic clerk held this program role for four years and noted three critical processes that helped facilitate the uptake of the program: 1) taking a patient-centred approach; and 2) building relationships and 3) reminder calls. In regard to a patient-centered approach, the clinic clerk noted:

I think the biggest factor into actually convincing people to come in was saying that the doctor had set aside this time specially for them and they could answer any questions that that patient may have. So I, would say, “Any questions you’ve ever had about your health, they’ll answer in this hour, and they want you to come in. It’s specifically for you” 2_Admin_4

They spoke further about how uptake became easier with time and the development of relationships with patients and caregivers:

The first year was hard for sure and it was, like, finding out who our patients were, and getting to know the patients, and who I call, because the numbers and the charts, you know, might be, they might be a worker but where is the phone number for the worker and where is it noted in the chart? … And then, as the years went on and I got to know the workers and the patients, and I could call, you know, so-and-so’s dad who has two IDD youth living, or, um, dependents living with him and say, “Oh, it’s me. They’re due again. It’s their yearly,” and so it was a lot easier as time went on, to when you form the relationship or even just know the right people to talk to, to schedule the appointment. 2_Admin_4

Reminder calls were also considered an important process for this population as the clerk noted: *“*it was noticeable if you didn’t do it. A lot of times there was no-shows, so [reminder calls were] an important thing to do for sure” 2_Admin_4.

The FHT’s health providers are engaged on an as needed basis in the care of adults with IDD and are not formally involved in the Health Check program. Nurse practitioners were involved in the initial program planning; however, their role in the organization changed and they were no longer involved. Processes around engaging a range of health providers were identified as not well developed and historically there have been challenges around their participation:

I’ve got to be honest with you, like, when I think about having non-physicians involved in this program and thinking about the life of the program, and I remember in the beginning when we were first starting, the, the physician-leads really wanted the interprofessional health providers to be involved. And, even made the process so that it was, like, almost dependent on the interprofessional health providers being involved. I do think there was some push-back from the interprofessional health providers at the time, that they didn’t want to be involved and they didn’t feel comfortable. 2_Admin_2

Limited involvement in this initiative and no formal processes to engage a range of health providers in the care of adults with IDD resulted in many health providers not working with adults with IDD in their practice and a general sentiment of feeling “less confident to deal with this population, yeah” 2_HCP_2. An environmental factor related to interprofessional primary care in Case two is the involvement of specialized IDD health providers that work specifically with this population at a local developmental service agency. Many of the FHT’s rostered adult patients with IDD are supported with on-going clinical care provided by these specialized IDD health providers (e.g. that include community nurses that function as care coordinators, occupational therapy, physical therapy, nutrition and psychological services). These health providers essentially function in a ‘shared care’ model with the physicians in the FHT. Consequently, many patients may not require the services of the interprofessional primary care team as readily as individuals without access to these specialized interprofessional services for adults with IDD. As one provider noted:

And the work of trying to organize that is, you know, very little for me at [Developmental Service Agency], because it, the system takes care of that and I get the benefit of all the information that, and advice and so on, that the other professionals generate. Whereas, for patients who don’t, aren’t hooked into that kind of system, it’s very difficult to organize services. They’re often not available. 2_HCP_3

**IDD community nurse position**.

The IDD community nurse position was piloted in 2007 and fully funded in spring of 2009 through an agreement with the Ministry of Community & Social Services (now the Ministry of Children, Community & Social Services). The IDD community nurse is co-located at the CHC and is a recognized member of the team, involved with all organizational functions and with access to all team resources (e.g. physical space, administrative support, access to shared EMR). The role of this IDD community nurse is to provide multiple primary care services to individuals with IDD in the region including assessment and screening, care coordination, system-navigation and health teaching for patients, as well as paid and unpaid caregivers. As part of the program, the nurse, in collaboration with a physician-lead from the CHC, conducts a weekly half-day IDD clinic. To be eligible to receive services from the nurse an individual must have a suspected or confirmed IDD. As per the CHC model, individuals do not have to be rostered with a health provider in the CHC to receive services; however, many individuals do receive their primary care there. Individuals can receive health care either at the clinic or in the community (e.g. place of residence) as required. Referrals can be formal or more informal through word of mouth.

The establishment of the IDD community nurse position was initiated by the closure of a large institution for individuals with IDD in the region and an identified gap in the knowledge of mainstream health providers in regard to this population. The position developed organically based on the identified needs of the community. The IDD community nurse role initially included a large degree of health teaching and capacity building within the community as developmental service workers took on additional responsibility for medication administration and health monitoring (traditional roles of health providers in institutional care). Although this remains a central role and the nurse continues to serve in a teaching and supportive capacity, many developmental service agencies have now established annual training processes to ensure caregivers have the competencies to complete these tasks.

The IDD community nurse position is in high demand and a request has been made to increase funding for additional hours. One of the reasons that the program is thought to be successful is because of being embedded within a CHC model of practice. As the IDD community nurse noted:

[Services] have to be dedicated. Yeah. So, how are you going to capture that in an environment other than this, right? Because who else does this? That’s why plunking this job in a CHC like this was the best happy happening. I think it just wasn’t super-planned but it, it was the best location -- I know, in the early days, there was a nurse that was hired, in the area to do it, and she was working from home and she was trying to connect with all of those providers…. [however] You are so isolated. I think having a doctor to do a designated clinic, it was the key. If I had to try and connect with all these other providers, it would be super-difficult to do, right? 5_HCP_1

The IDD community nurse is recognized as a source of expertise for this population within the team; the nurse is always engaged in the event of a new or suspected patient with IDD and health providers will regularly consult with the nurse regarding system navigation and resources for this population. Although of interest to the organization, the IDD community nurse position and the collaborative IDD clinic are not currently evaluated or formally identified in organizational quality improvement initiatives. In addition, the CHC has not gone through a formal process to identify individuals with IDD within the organization; largely due to the fact that all patients are known to, and followed by, the IDD community nurse. This program of care is heavily reliant on the IDD community nurse and the largest potential risk that has been reported in regard to this program is its sustainability both from a funding and human resources perspective.

The nurse is the primary contact for all individuals in the IDD program and will support caregivers in triaging health situations to avoid unnecessary hospitalizations. The nurse also acts in a care coordinator capacity and assists with advocating for needed resources and system navigation, often attending other medical appointments with the patient or assisting to prepare the patient and caregiver with questions to ask. Formal Health Checks are not mandatory; however, most group home clients do receive an annual physical.

The physician and nurse have established informal care coordination processes for this clinic and will work collaboratively to manage patient issues. For all new patients of the collaborative IDD clinic, the nurse gathers all historical medical and social records, identifies caregivers and social supports and begins a medical record. The nurse assists with scheduling for the clinic and prepares all documentation in advance, as well as completes post visit follow up and monitoring. The IDD community nurse will assist in scheduling (and accompany if needed) individuals to other provider appointments. The clinic clerk will assist in scheduling formal appointments, especially with the physician, collaborative clinic or member of the interprofessional team. Reminder calls are provided.

There are no formal processes to engage health providers in the care of this group, above and beyond the IDD community nurse and physician. Other health providers on the team are engaged on an as needed basis for issues such as counselling (social work) and healthy eating (dietitian). The process for engagement appears to be dynamic and facilitated by co-location as the IDD community nurse described:

It’s just here it’s like, ‘Oh, we got a social worker here.’ Like, ‘Oh, I just saw the dietitian in the hallway, we talked.’    And if we’re in an appointment and, and Dr. says, ‘Yeah, I think there’d be some really good value to you seeing the dietitian.’ I often bring things up, too. It’s like, ‘Hey, Dr., what do you think if we access these services whatever we like, boom, boom, boom, make it here and we make it happen. 5_HCP_1
